# Medication non-adherence and therapeutic inertia independently contribute to poor disease control for cardiometabolic diseases

**DOI:** 10.1038/s41598-022-21916-8

**Published:** 2022-11-07

**Authors:** Xiaowei Yan, Satish Mudiganti, Hannah Husby, Andrew Hudnut, Madina Gbotoe, J. B. Jones

**Affiliations:** 1 Sutter Health, Center for Health Systems Research, Walnut Creek, CA 94596 USA; 2 Sutter Medical Group, Elk Grove, CA 95758 USA

**Keywords:** Health services, Public health, Risk factors

## Abstract

Poorly controlled cardiometabolic biometric health gap measures [e.g.,uncontrolled blood pressure (BP), HbA1c, and low-density lipoprotein cholesterol (LDL-C)] are mediated by medication adherence and clinician-level therapeutic inertia (TI). The study of comparing relative contribution of these two factors to disease control is lacking. We conducted a retrospective cohort study using 7 years of longitudinal electronic health records (EHR) from primary care cardiometabolic patients who were 35 years or older. Cox-regression modeling was applied to estimate how baseline proportion of days covered (PDC) and TI were associated with cardiometabolic related health gap closure. 92,766 patients were included in the analysis, among which 89.9%, 85.8%, and 73.3% closed a BP, HbA1c, or LDL-C gap, respectively, with median days to gap closure ranging from 223 to 408 days. Patients who did not retrieve a medication were the least likely to achieve biometric control, particularly for LDL-C (HR = 0.58, 95% CI: 0.55–0.60). TI or uncertainty of TI was associated with a high risk of health gap persistence, particularly for LDL-C (HR ranges 0.46–0.48). Both poor medication adherence and TI are independently associated with persistent health gaps, and TI has a much higher impact on disease control compared to medication adherence, implying disease management strategies should prioritize reducing TI.

Cardiometabolic conditions, including hypertension, dyslipidemia and diabetes, are major risk factors for cardiovascular disease and a leading cause of mortality worldwide. Management of these conditions is a dominant focus in primary care^1,2^. Though advances in pharmacotherapy have led to guidelines that articulate how to treat and manage cardiometabolic conditions, less than half of patients achieve control for related clinical measures (e.g., blood pressure (BP), low-density lipoprotein cholesterol (LDL-C), HbA1c)^3–6^, which substantially increases the risk of preventable morbidity and mortality^7–11^.

Achieving biometric treatment goals is mediated by clinician (e.g., knowledge, communication, familiarity with guidelines) and patient-level factors (e.g., environment, lifestyle, genetics), among which two key actionable components are widely studied: clinician-level therapeutic inertia (TI) and patient-level medication adherence. Studies have shown that each of these components contribute to disease control and future risk of cardiovascular complications^12–15^. TI, defined as the failure to initiate or titrate therapy according to clinical guidelines when treatment goals are not achieved, is common^16–22^. Medication adherence, defined as the extent to which a person takes medications recommended by a health care professional, also plays a critical role in cardiometabolic-related biometric control. A prior study suggested that each 10% incremental increase in medication adherence led to a 0.16%-point reduction in HbA1c^23^. However, adherence to cardiometabolic-related medications is still suboptimal and varies significantly by medication type and underlying conditions^24–31^ with the lowest adherence in statins (25–40%)^32^. Most previous studies focus on assessing the impact of a single component on disease control, whereas studies that simultaneously study both TI and adherence are still lacking^34^. Readily available medication dispense data in electronic health records (EHRs) makes it possible to derive measures of medication adherence to assess how much these factors contribute to biometric measure control.

Many studies have focused on delayed medication initiation or intensification and the impact on clinical outcomes for cardiometabolic conditions^35–38^. However, few studies report the time to achieve disease control in real-world practice. Among adult patients with a cardiometabolic condition, more than half had two or more cardiometabolic diseases^34,39^, denoted as multimorbidity, implying a potential common underlying pathophysiological mechanism. The interplay of risk factors for cardiovascular disease suggests that cardiometabolic conditions and risk factors should be managed together^40,41^. There is emerging evidence on elevated cardiometabolic risk burden in people with multimorbidity, and challenges to achieving disease control, such as polypharmacy^42–44^. It is critical to understand medication adherence, TI and their association with disease control in populations with polypharmacy and multimorbidity.

We use longitudinal EHR data from patients with at least one cardiometabolic condition to examine the association between TI, medication adherence, and biometric control for each condition and multimorbid patients.

## Methods

### Study population and study design

This retrospective cohort study focused on the primary care population at Sutter Health, a large healthcare system in northern California, serving ~ 3 million patients, including about 1.1 million primary care patients, through 272 outpatient primary care clinics and approximately 1700 primary care physicians (PCPs). Sutter patients > 35 years old and diagnosed with at least one cardiometabolic condition were eligible for this study. Details on the diagnosis criteria for each cardiometabolic condition are described elsewhere^34^, and EHR data back to 2010 were used to check the earliest diagnosis for each cardiometabolic condition.

**Figure 1:**
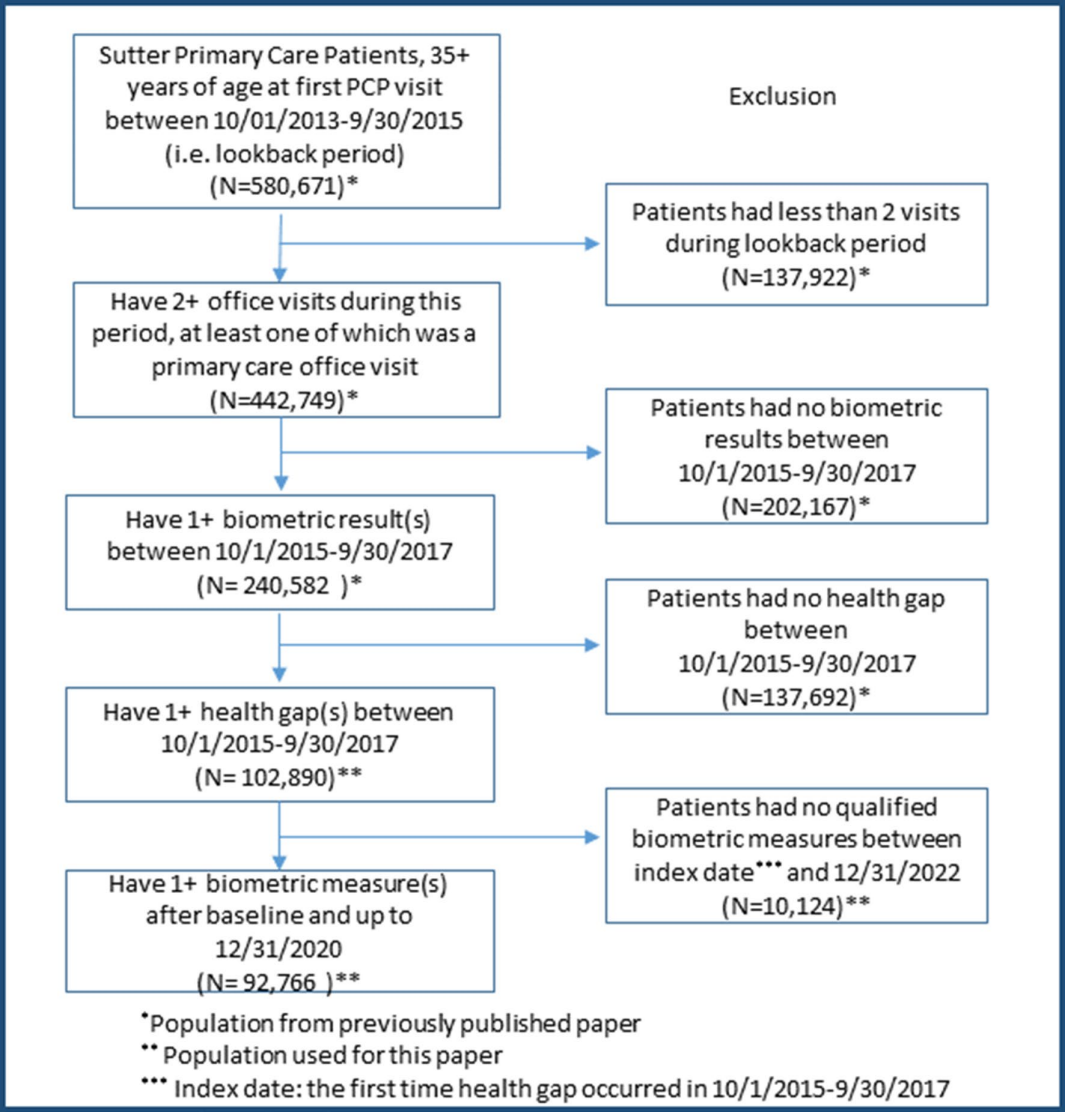
Flowchart for study cohort identification. Data flowchart for patients meeting study eligibility criteria and cohort identification.

We applied similar criteria as in our previous publication to capture elligble patients, where patients were included in this study if they had: 1) at least one PCP visit during the time period from 10/1/2013–9/30/2015 (i.e., eligibility period) to make sure patients were actively seeking primary care at Sutter; 2) at least one health gap (e.g., elevated BP, elevated HbA1c, or elevated LDL-C) during 10/01/2015–09/30/2017, the health gap assessment period; and 3) SureScripts medication dispense data in health gap assessment period. (Fig. 1).

**Figure 2:**
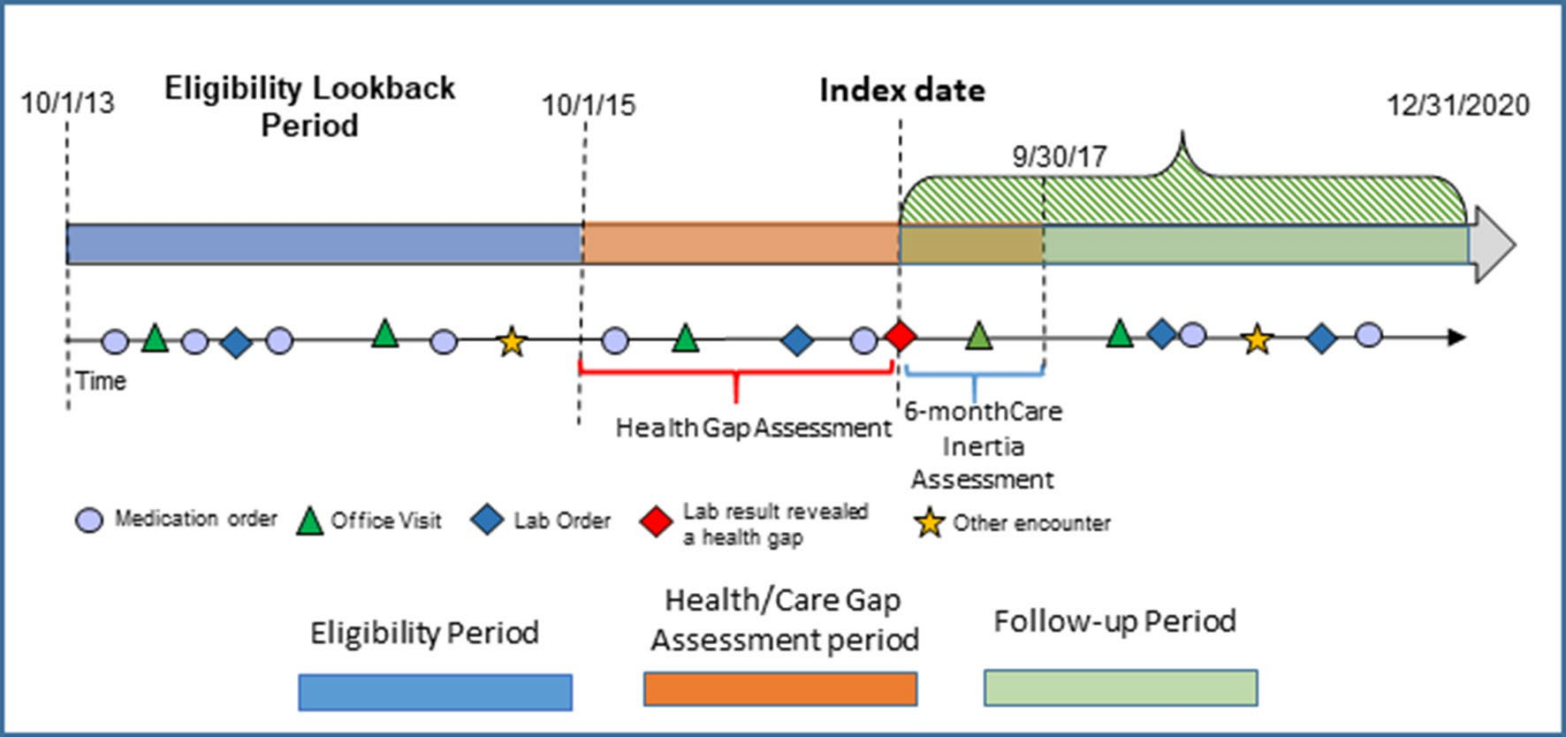
Retrospective use of EHR data to identify eligible primary care patients, health gaps, clinical inertia and follow-up period. Study eligibility criteria timeline depicting how primary care patients, health gaps, clinical inertia and follow up period were identified**.**

The detailed criteria for defining health gaps are shown in Table 1. Two elevated measures at two consecutive clinical encounters were required for BP, and one was required for HbA1c and LDL-C^34^. The index date for each biometric measure was defined as the date when the first elevated measure was observed within the health gap assessment period. TI was identified within the 6-month period after the index date, and the approach to using EHR data to identify TI was described elsewhere^34^ In this study, the operational criteria for TI are found in Table 1. In our previously published work, the study period ended at 9/30/2017; to study persistence of care gaps, in this study we followed patients until 12/31/2020 (See Fig. 2), and we further expanded our inclusion criteria to require at least one qualified biometric measure in the follow-up period.

**Table 1:** Operational criteria for defining health gaps, health gap closure and clinical inertia.

Health gap criteria*	Achievement of disease control
Diastolic BP ≥ 90 mmHg OR systolic BP ≥ 140 mmHg in two consecutive clinical encounters	Diastolic BP < 90 mmHg AND systolic BP < 140 mmHg in two consecutive clinical encounters
If CHD 10-year risk† > 20% then LDL health gap was defined as ≥ 100 mg/dL	If CHD 10-year risk† > 20% then LDL health gap was defined as < 100 mg/dL
If there were 2 + risk factors† or the 10-year risk ≤ 20% then the LDL health gap was defined as ≥ 130 mg/dL	If there were 2 + risk factors† or the 10-year risk ≤ 20% then the LDL health gap was defined as < 130 mg/dL
If there were 0–1 risk factors then the LDL health gap was defined as ≥ 160 mg/dL	If there were 0–1 risk factors then the LDL health gap was defined as < 160 mg/dL
HgA1c ≥ 8.0%	HgA1c < 8.0%
Clinical Inertia Status	Criteria of Actions Taken to Close Therapeutic Inertia
No^‡^	Treatment is initiated or intensified by increasing the dose of at least one medication or by adding a second medication to the existing regimen. Existing medication was reordered^§^,
Yes	Medication is the same as the pre-health gap medication(s), or no medication was prescribed in the post health gap period
Uncertain	Total number of medications is the same, part of the medication regimen has been changed, and for medications that are not changed doses are the same

*—Criteria are consistent with the American College of Cardiology (ACC)/American Heart Association (AHA) guidelines. †—Factors used to estimate coronary heart disease (CHD) risk: age, total cholesterol, smoking status, HDL, systolic BP, and antihypertensive treatment. The formula can be found in ATP III guidelines. ‡—Assumes that the physician discussed adherence with the patient, but the patient may have also acted on their own to improve treatment adherence. §,—Assumes that the physician recognized the patient self-care gap and acted to close the care gap with a new prescription order with or without contacting the patient.

### Data resources

Seven years of structured EHR data (i.e., 10/1/2013–12/31/2020) were extracted and used for this study. We used Surescripts medication dispense data to derive medication adherence.

Medication adherence was assessed using the proportion of days covered (PDC)^45^ and was calculated as days covered by the filled prescription period divided by the number of days between the first fill date within a year before the index date and the index date. We defined medication adherent as PDC ≥ 80% and not adherent as PDC < 80%^47^, and “no medication retrieved” if there were no medication dispense data for a given medication order.

As shown in Table 1, TI was defined as the absence of any EHR record of a medication order or intensification (e.g., increased dose, add medications) for any patient with a health gap. We created a category named “uncertain” to denote cases where medication intensification or titration was uncertain based on the structured data available.

### Study outcome

The primary outcome of the study is the first time to achieve biometric control during the follow-up period (i.e., from index date to 12/31/2020). Each biometric measure that never met disease control criteria are censored at the end of the study period (i.e., 12/31/2020).

### Statistical analysis

Using longitudinal EHR, we estimated the following: (1) prevalence of health gap closure and TI during the follow-up period, (2) length of time to close a health gap, and (3) association between disease control and baseline medication adherence and TI.

Descriptive statistics were reported, categorized by number and type of health gaps at baseline, and included demographics, BMI, disease burden (measured by Charlson Comorbidity Index (CCI))^46^, baseline medication adherence, baseline biometric measures, TI status, follow-up length, and percentage and length of time to health gap closure (Table 2). Separate summary statistics were calculated for each cardiometabolic condition, stratified by health gap closure status (Supplementary Table S1).

**Table 2:** Baseline Characteristics for patients with at least one biometric health gap at baseline, stratified by number and types of health gaps at baseline.

Patient demographics and Other factors	Baseline Health Gap Combinations						
	HbA1c Gap only (N = 8302)	BP gap only (N = 41,606)	LDL Gap only (N = 22,843)	HbA1c and BP gap (N = 3666)	LDL and BP gap (N = 11,714)	HbA1c and LDL gap (N = 2534)	All three gaps (N = 2101)
**Age group**							
34–39	7.4%	4.9%	7.0%	3.7%	2.2%	9.6%	4.6%
40–49	22.8%	14.7%	23.9%	15.1%	11.8%	33.1%	20.0%
50–64	29.5%	21.3%	32.8%	25.5%	25.7%	29.7%	28.7%
65–79	24.5%	27.4%	23.6%	31.3%	30.7%	18.8%	28.8%
80 +	15.7%	31.7%	12.8%	24.4%	29.6%	8.9%	17.9%
**Race/ethnicity**							
Hispanic	17.3%	9.0%	9.3%	16.8%	9.7%	18.9%	17.0%
NH-Asian	16.3%	9.6%	17.2%	12.6%	10.5%	18.9%	12.5%
Black	5.0%	4.3%	2.8%	6.8%	4.7%	5.1%	6.8%
NH-Other	13.6%	10.7%	12.8%	13.2%	11.2%	13.8%	12.4%
NH-White	47.9%	66.4%	58.0%	50.7%	63.9%	43.3%	51.2%
**Sex**							
Female	43.3%	56.0%	60.2%	46.0%	63.8%	48.9%	52.5%
**BMI**							
< 25	12.8%	25.7%	26.5%	10.4%	21.4%	12.9%	10.2%
25–29	29.3%	35.7%	39.8%	26.3%	36.8%	31.9%	26.7%
30–34	28.3%	22.0%	21.2%	29.5%	24.8%	28.1%	30.9%
35 +	29.1%	16.2%	12.1%	33.5%	16.6%	26.9%	31.8%
Missing	0.6%	0.4%	0.4%	0.4%	0.3%	0.3%	0.3%
**Smoking status**							
Never	57.2%	55.8%	63.5%	53.6%	57.1%	59.6%	54.9%
Passive/quit	35.9%	38.2%	30.4%	40.2%	37.2%	33.3%	37.9%
Yes	6.9%	6.0%	6.1%	6.2%	5.7%	7.1%	7.2%
**Charlson score**							
0	45.1%	60.2%	73.8%	31.0%	59.9%	52.5%	34.0%
1–2	44.8%	33.0%	23.1%	54.0%	33.8%	38.8%	52.6%
3 +	10.1%	6.7%	3.1%	15.0%	6.3%	8.8%	13.4%
**Number of cardio-metabolic conditions**							
1	3.5%	35.4%	34.6%	0%	0%	0%	0%
2	22.7%	49.6%	45.9%	15.3%	68.0%	21.6%	0%
3	73.8%	14.9%	19.6%	84.7%	32.0%	78.4%	100%
Baseline LDL level			154 (30.1)		143.1 (28.0)	126.7 (24.9)	127.4 (25.6)
Baseline Systolic BP/ Diastolic BP		151.2 (12.4)/82.1 (10.7)		152.5 (12.2)/80.3 (10.9)	151.8 (12.7)/82.4 (10.5)		152.7 (13.4)/82.1 (10.6)
Baseline HbA1c	9.2 (1.4)			9.2 (1.3)		9.4 (1.4)	9.3 (1.4)
**Diabetes PDC status**							
Not adherent	20.7%	–	–	24.3%	–	24.7%	26.4%
Adherent	39.0%	–	–	39.1%	–	33.9%	35.4%
No medication retrieved	40.4%	–	–	36.6%	–	41.3%	38.2%
**Hypertension PDC status**							
Not adherent	–	12.0%	-	18.8%	14.9%	–	23.2%
Adherent	–	38.1%	-	46.3%	38.4%	–	40.5%
No medication retrieved	–	49.9%	-	34.9%	46.7%	–	36.3%
**LDL PDC status**							
Not adherent	–	–	7.0%	–	8.7%	12.7%	14.4%
Adherent	–	–	10.9%	–	16.0%	21.6%	23.4%
No medication retrieved	–	–	82.1%	–	75.3%	65.8%	62.2%
**Diabetes clinical inertia**							
No	42.6%	–	–	40.8%	–	45.9%	43.8%
Yes	40.8%	–	–	40.0%	–	34.8%	35.9%
uncertain	16.6%	–	–	19.3%	–	19.3%	20.3%
**Hypertension clinical inertia**							
No	–	32.3%	–	39.1%	32.2%	–	38.7%
Yes	–	18.1%	–	21.0%	19.4%	–	19.9%
uncertain	–	49.7%	–	39.8%	48.4%	–	41.4%
**LDL clinical inertia**							
No	–	–	25.3%	–	26.3%	36.6%	34.5%
Yes	–	–	7.7%	–	11.5%	14.0%	15.8%
uncertain	–	–	67.1%	–	62.2%	49.4%	49.7%
**Diabetes before dyslipidemia**							
Yes	44.1%	11.1%	32.6%	45.3%	34.3%	50.9%	50.4%
**Diabetes before hypertension**							
Yes	39.5%	28.9%	10.0%	39.6%	23.0%	36.6%	39.7%
**Length with dyslipidemia**							
< 1 year	–	–	4.8%	–	3.4%	2.6%	2.2%
1–4 years	–	–	67.4%	–	67.4%	65.3%	63.4%
5 + years	–	–	27.8%	–	29.2%	32.1%	34.5%
**Length with hypertension**							
< 1 year	–	3.2%	–	0.1%	2.2%		0.1%
1–4 years	–	46.0%	–	38.8%	44.6%		40.7%
5 + years	–	50.8%	–	61.2%	53.2%		59.3%
**Length with diabetes**							
< 1 year	5.0%	–	–	2.9%	–	4.7%	4.1%
1–4 years	46.2%	–	–	44.9%	–	49.2%	47.2%
5 + years	48.8%	–	–	52.2%	–	46.1%	48.7%
**Outcome measure**							
Time to close LDL gap (n, medium, IQR)			16,798 (73.5%) 406 (219–756)		8318 (71.0%) 418 (216–785)	1933 (76.3%) 395 (189–716)	1670 (79.5%) 397 (188–699)
Time to close BP gap (n, medium, IQR)		37,327 (89.7%) 224 (93–491)		3407 (92.9%) 190 (84–429)	10,458 (89.3%) 238 (96–525)		1920 (91.4%) 201 (84–492)
Time to close HbA1c gap (n, medium, IQR)	7187 (86.6%) 220 (117–483)			3196, (87.2%) 225 (116–512)		2087 (82.4%) 246 (119–516)	1782 (84.8%) 219 (113–526)

For each cardiometabolic condition, we assessed the association between baseline medication adherence, TI, and time to achieve disease control using Cox regression models. If the proportional hazard assumption is valid for each covariate, the Cox model is reported adjusting for baseline characteristics shown in Table 3. Hazard ratio (HR) and 95% CI were reported for medication adherence, and for TI, adjusting for baseline characteristics, including age groups, gender, race/ethnicity, body mass index (BMI), Charlson comorbidity index (CCI), smoking status, alcohol status, insurance, number of office visits within 10/1/2015–9/30/2017, length of history with corresponding cardiometaolic condition (< 1 year, 1–4 years, and 5 + years), number of cardiometablic conditions and corresponding baseline biometric value.

**Table 3:** Hazard Ratio estimate from Cox regression models for each biometric measure during 5 years of follow-up*

Variable	HR (95%CI)		
	BP control	LDL-C control	HbA1c control
**Therapeutic inertia**			
No	Ref	Ref	Ref
Yes	0.90 (0.88–0.93)	0.48 (0.46–0.50)	0.91 (0.87–0.95)
Uncertain	0.95 (0.93–0.96)	0.46 (0.45–0.47)	0.88 (0.80–0.96)
**Baseline medication adherence**			
Adherent	Ref	Ref	Ref
Not Adherent	1.0 (0.97–1.03)	0.98 (0.93–1.03)	0.92 (0.84–1.0)
Not Retrieved	1.01 (0.99–1.03)	0.58 (0.55–0.60)	0.93 (0.88–0.97)
**Number of cardiometabolic conditions**			
1	Ref	Ref	Ref
2	1.10 (1.07–1.12)	0.47 (0.44–0.50)	1.16 (1.01–1.33)
3	1.18 (1.15–1.21)	0.23 (0.21–0.24)	1.09 (0.96–1.25)
**Length with disease**			
< 1	Ref	Ref	Ref
1–4	0.71 (0.67–0.75)	0.85 (0.80–0.91)	0.44 (0.41–0.48)
5 +	0.70 (0.67–0.74)	1.04 (0.94–1.07)	0.51 (0.47–0.56)

*In addition to above variables, we further controlled additional baseline characteristics, including age groups, gender, race/ethnicity, body mass index (BMI), Charlson comorbidity index (CCI), smoking status, alcohol status, insurance, number of office visits between 10/1/2015–9/30/2017, and corresponding baseline biometric value.

We further conducted a set of analyses to examine whether patients with more than one health gap at baseline are more or less likely to achieve disease control. To that end, for each oucome, a categorical variable that indicated specific combinations of health gaps was included in the Cox model, and we used the model to assess the relative risk (i.e., HR) for the outcome of achieving control between patients with more than two baseline health gaps compared to those with a single health gap. For example, for BP control as the outcome, patients who had baseline BP gap were included, and a categorical variable that indicated the baseline health gap combinations, such as BP gap only, both BP and LDL gaps, both BP and HbA1c gaps, and all three gaps, was further included in the model, along with all baseline characteristics, and BP gap only was taken as the reference group.

To quantify the relative importance of medication adherence and TI variables in impacting biometric control, we adopted the method proposed by Korn and Simon^48^ to calculate Somers’D, representing the correlation between the test variable and survival outcomes (i.e., biometric measure control status). The relative difference of Somers’D between the full (including all variables in the Cox regression model) and reduced model (medication adherence or TI variables removed) was an approximate measure of variation explained by the medication adherence variable.

All methods were performed in accordance with relevant guideline and regulartions. This study was reviewed and approved by the Sutter Health Institutional Review Board (IRB). As a retrospective study, IRB has approved to waive the need for informed consent to patients.

## Results

### The study population and prevalence of health gap at baseline

Among 92,766 patients who met the inclusion criteria, 63.7% (59,087), 17.9% (16,603) and 42.2% of patients had a BP, HbA1c, and LDL-C gap respectively (Supplemental Table S1). Patients with a BP gap were oldest compared to those with an HbA1c or LDL gap at baseline, female were likely to have LDL gap (60.1%), but a smaller proportion had an HbA1c gap (45.9%). Patients with an HbA1c gap had more disease burden (i.e., Charlson comorbitidity score 3 +) (11.4% vs. ≤ 7.4%).

The average follow-up time is 5.1 (SD = 0.6) years, within which 89.9% had their BP health gap closed with the median time to closure being 223 days (Interquartile or IQR = 92–494). 85.8% had a HbA1c gap closed with the median time to gap closure being 223 days (IQR = 117–500). A significantly lower percentage of patients (73.8%) had an LDL-C gap closed, and it took significantly longer to close the gap (median = 408 days, IQR = 214–758) (Supplemental Table S1).

As shown in Table 2, 8.9%, 44.9%, and 24.6% had a single HbA1c, BP, or LDL-C gap, respectively. Slightly over 19% of patients had two health gaps, and 2.3% had all three gaps. Patients with a BP gap only were older and patients with both LDL-C and HbA1c gaps were the youngest. Patients with both HbA1c and BP gaps had the highest disease burden (15% had 3 + comorbidities), while the lowest disease burden was observed for patients with an LDL-C gap only (3%).

Among patients who had a BP gap only (N = 41,606), 89.7% had BP gap closed, similar to patients who had both LDL and BP gaps (89.3%), but lower than those who had both HbA1c gap and BP gap (92.9%) (Table 2). Patients with both HbA1c and BP gaps also had highest proportion of HbA1c gaps closed (87.2%), a slightly higher proportion than those who only had an HbA1c gap, and significantly higher than those who had both an LDL and HbA1c gap at baseline (82.4%). Among patients who had LDL gaps, the highest LDL gap closure occurred in patients with all three health gaps at baseline (79.5%), and lowest LDL gap closure appeared to be in patients with both LDL and BP gaps (71.0%) (Table 2).

### Prevalence of medication adherence and therapeutic inertia

Patients were likely to adhere to anti-hypertensive medications (> 38%) while almost half of patients did not retrieve their medication (35%-50%). Only 34–39% of patients with a HbA1c gap adhered to their anti-diabetic medication, and the highest adherence was observed in patients with both HbA1c and BP gaps (39%). Medication adherence was lowest (11–23%) for lipid-lowering medications (mainly statins) and a significantly greater proportion of patients with an LDL-C gap did not retrieve their statins, ranging from 62 to 82%.

TI existed for 35–41% of patients with a HbA1c gap, significantly higher than that for patients with BP or LDL-C gaps (< 21% for BP gap, and < 16% for LDL-C gap). However, TI status was uncertain for a significant number of patients with an LDL-C gap (49–67%) since the statin intensity or dose did not change before and after the LDL-C gap. (Table 2).

### Association between medication adherence and therapeutic inertia and disease control

The Kaplan–Meier survival curves (Supplemental Fig. S1) revealed that different medication adherence levels or TI categories varied significantly on LDL control, but to a lesser degree on HbA1c control, and had the least impact on BP control. The “no mediation retrieved” category had the poorest LDL control. For TI, “uncertain” TI had the lowest likelihood of LDL control, followed by definite TI.

The above findings did not change meaningfully after adjusting for baseline characteristics. The adjusted HR for LDL control for those who had TI was 0.48 (95% CI): 0.46–0.50, “no TI” as the reference) and was 0.46 (95% CI: 0.45–0.47) for the uncertain TI category. Among patients with a HbA1c gap, the HR for those who had TI was 0.91 (0.87–0.95) and 0.88 (0.80–0.96) for the uncertain group. This was similar to the association between TI and BP control (Table 3).

The association between baseline medication adherence and gap closure status was strongest for LDL “medication not retrieved” with a HR of 0.58 (0.55–0.60) compared to adherent patients, and to a lesser degree for HbA1c control (HR 0.93 (0.88–0.97)), and no statistical significance was observed for BP control (P > 0.20) (Table 3).

Patients with multiple health gaps were generally associated with poorer disease control compared to those who only had a single health gap (Supplemental Table S2) with a HR ranging from 0.91 to 0.98, except for LDL-C control, in which, patients with all three gaps had better LDL control than those who only had an LDL gap.

### Relative attribution of medication adherence and therapeutic interia to disease control

Finally, Table 4 shows a statistical measure for the variation explained by TI or medication adherence for each biometric. For HbA1c, the full model explained 21.4% of the variation. After removing the TI variable, the reduced model explained 21.2% of the variation, implying that the variation explained by TI is about 0.2%. Similarly, we found medication adherence has no significant contribution in predicting HbA1c control. TI and medication adherence did not explain much of the variation in BP control. However, TI explained the most variation in predicting LDL control, in which 9.6% of the variation was explained by the TI variable alone, and 2.6% by medication adherence.

**Table 4:** Variation explained by proportion of days covered (PDC) and therapeutic inertia:

Outcome	Somers’ D for full model	Somers’ D for reduced model without Therapeutic inertia variable	Variation explained by Therapeutic inertia* (%)	Somers’ D for reduced model without Medication adherence variable	Variation explained by Medication adherence* (%)
HbA1c	0.214	0.212	0.2	0.214	0
LDL-C	0.318	0.222	9.6	0.292	2.6
BP	0.194	0.190	0.4	0.194	0

*Difference between Somers’D for full model and reduced model.

## Discussion

### Summary of learnings

In this study, we follow primary care patients who had poorly controlled BP, HbA1c, or LDL-C at baseline for more than 5 years and simultaneously assessed the impact of TI and medication adherence on health gap persistence. Though both TI and poor medication adherence are highly associated with persistence of poor biometric control, our study found that TI has a much stronger impact than medication adherence, particularly for LDL, in which TI explained three times more variation than medication adherence. Furthermore, it takes much longer to achieve LDL control (> 400 days), compared to about 200–240 days to achieve HbA1c or BP control in this real-world setting. Among patients with multiple health gaps, if one is LDL, patients are less likely to achieve disease control for other biometric measures. To our knowledge, it is the first study to quantify relative attribution of medication adherence and TI to BP, HbA1c and LDL-C control in a large cohort. We are able to differentiate the primary non-adherence (patients who did not retrieve the medication) from non-adherence (0 < PDC < 80%), which captures a complete picture of patient adherence to medications compared to using pharmacy claims data only, in which only non-adherence can be derived, and primary non-adherence is likely to be completely missing.

### Compare to other studies

In our previously pubished work we have shown that EHR data can be used to capture medication adherence and TI^34^, and in this study, we further investigated how the medication adherence and TI impact long-term disease control.

TI is very common in managing cardiometabolic patients. Past studies show large variation in TI in controlling HbA1c, lipids, and BP, ranging from 30 to 80%, varying by study cohort, ASCVD risk, medication regimens, geographic region, and study design^22,36,49–52^. In this study, we investigate additional details on medication prescription patterns, and further breakdown TI into three categories “No TI,” “Definite TI,” and “Uncertain TI.” The “Definite TI” group accounts for about 10–43% of out-of-control cardiometabolic related measures. However, TI status cannot be discerned due to inadequate information^34^ for a majority of out-of-control dyslipidemia patients (~ 64%) and to a lesser degree of poorly controlled hypertensive or diabetes patients (49% and 18%). We define “uncertain” as partially due to limitations in structured EHR data capturing reasons for not initiating or intensifying treatment and to a large proportion of switched medications that are hard to conclude as intensification. Regardless of reasons for “uncertain,” we have found that patients with uncertain TI had the poorest disease control, even when compared to patients with definite TI. Strikingly, it takes 1.6 times longer time to achieve LDL control for patients with uncertain TI compared to no TI group (485 days vs. 296 days). It suggests that disease control can be significantly improved if clinicians take clinical actions, such as reorders, initiations, or intensifications.

Poor medication adherence is a well-known factor associated with poor clinical outcomes^53–57^. It has been reported that adherence to antihypertensive medication has improved in the past decade to as high as 75% to 90% partially due to BP control being built into quality metrics in most healthcare systems^26,27,58–60^, but still has large variation among different study populations^59,60^. Statin adherence remains suboptimal, varying significantly by comorbidities, ranging from 36% for patients without coronary heart disease or diabetes, to 64% for patients with myocardial infarction^33^. In our study, medication adherence for those with all three cardiometabolic conditions is lower than previously published studies^59^. The use of dispense data only for estimating adherence, the possibility of missing refill data^59^ or instances where patients fail to fill prescribed medications may explain the lower adherence observed in our study, where the latter patients were not included in the denominator in most previous studies. We identify a large proportion of patients that did not retrieve their prescribed medication, ranging from 48% for antihypertensive medication to as high as 62%-82% for statins. Interestingly, we did not observe that poor medication adherence is associated with worse BP or HbA1c control, only that “failure to retrieve medication” is significantly associated with persistence of poor LDL or HbA1c control.

Our study reveals that TI plays a much more crucial role in disease control compared to medication adherence, particularly for LDL. To our knowledge, this is the first study to compare the relative contribution of risk factors in disease control, and TI explained three times more variation in LDL control than medication adherence, highlighting that efforts to addressing TI might be more effective in managing LDL. Clinician and patient education, team-based care, and population health management strategies, such as system-wide quality improvement and/or variation reduction and clinical decision support systems, may be effective in reducing TI^51^. Moreover, studies have indicated that not only is TI common among patients with cardiometabolic conditions, the actions to intensify medications are delayed significantly from the time a biometric measure is elevated. On average, a 1.5-year to 5-year delay is observed in antihyperglycemic treatment^38^, which further highlights the urgency to act.

Interestingly, our study showed that patients with multiple cardiometabolic conditions were likely to achieve better BP or HbA1c control compared to patients with a single condition. This result implies that BP and HbA1c can be managed together^61–63^.

In real-world clinical settings, PCPs have many competing priorities in the limited clinical time they have with patients^64^. Population health management becomes a common approach in healthcare to reach out to high risk patients and guide them to enhanced care. Prioritization of clinical management is challenging, particularly for patients with multiple comorbidities. Our analysis shows that BP or LDL health gaps are more likely to be persistent in patients with multiple health gaps compared to those with a single health gap (supplemental S2). In population health management efforts, in addition to using ASCVD risk score to identify high risk patients, prioritization of outreach based on the number of health gaps may be an intuitive and efficient way to achieve better disease control for patients with multiple cardiometabolic conditions..

## Limitations

There are limitations to this study. First, it is based on a single healthcare system. However, the system serves a diverse population, in which race/ethnic minorities account for about 40% of the population and is representative of a growing minority population in the US. Second, medication dispense data may not capture all refill data or reflect actual patient use. However, dispense data are an inexpensive, easy approach to capture medication adherence for a population of patients, and provide objective information for clinicians to engage with patients. Third, in this study we did not differentiate the primary prevention of atherosclerotic cardiovascular disease (ASCVD) from secondary prevention. In clinical practice, each ASCVD risk factor (i.e., high blood pressure, high LDL-C, diabetes, etc.) is usually monitored and managed individually and PCPs may pay more attention to secondary prevention than primary prevention. Future studies can separate out and study the persistence of health gaps in each sector. Fourth, we did not consider white-coat effect on BP measurement. It is challenging to differentiate white-coat hypertension from other hypertension in the EHR. However, studies have shown that patients with white-coat hypertension, left untreated, had elevated risk for cardiovascular disease. Improving medication adherence and reducing TI might also be applicable to white-coat hypertension. Further study to drill down intos different type of hypertension is needed. Finally, we only used structured EHR data to identify TI without understanding underlying reasons. We may have attributed a patient’s decision to decline medication intensification to a clinician and thus overestimated clinical inertia. Future studies can explore the development and implementation of natural language processing algorithms to identify reasons for TI from progress notes. Despite these limitations, the study is based on a racially and ethnically diverse population and uses easily available data that ensures the possible extrapolation and replication of the work.

TI has a larger impact on disease control compared to medication adherence, particularly for LDL control, in which the time to achieve disease control is significantly longer (> 400 days) than to achieve BP or HbA1c control (< 250 days). Patients with multiple health gaps were less likely to achieve disease control compared to patients with a single health gap, which may lead to a higher risk for ASCVD. These findings suggest that clinical management should be prioritized based on number of health gaps, rather than merely on number of comorbidities.

## Clinical implications and future research

More attention is needed on therapeutic inertia regarding lipid, glycemic and blood pressure management among patients with cardiometabolic conditions, particularly for patients with multiple health gaps given it may take longer to close a health gap. Actions to close health gaps might be multi-dimensional, focused not only on physician’s actions, but also those of other health professionals including population health managers and pharmacists who may work together to promote patient education, self-management, and well-being, which requires health systems to implement effect policies and care delivery models to delivery more efficient and timely care.

Although our study has highlighted the importance of TI in persistence of health gap, there are still important questions that merit additional research, such as the underlying causes of TI, whether unconcious bias plays a role in physicians’ prescription patterns, the reasons why older patients have more TI for BP control, the reaons why women have more TI for lipid control, and whether unconcious bias is a reason that treating BP in older patients does not seem to help. Answering these questions may require mixed methods to collect quantititive data from both structured data sources and, using methods such as natural language processing (NLP), to extract insights from unstructured free text data (e.g., progress notes, email or phone conversation with patients), as well as qualitative methods such as interviewing PCPs and patients.

## Supplementary Information

Supplementary Information 1.

Supplementary Information 2.

## Data Availability

The datasets generated during and/or analyzed during the current study are not publicly available due to patient privacy considerations that prohibit the sharing of patient-level data but data (in summary statistics, non-identifiable aggregated form) are available from the corresponding author on reasonable request.
