# Supplementary figures and images for: Medication non-adherence and therapeutic inertia independently contribute to poor disease control for cardiometabolic diseases
Source: Sci Rep. 2022 Nov 7;12:18936. doi: 10.1038/s41598-022-21916-8 (PMC9640683; doi:10.1038/s41598-022-21916-8)

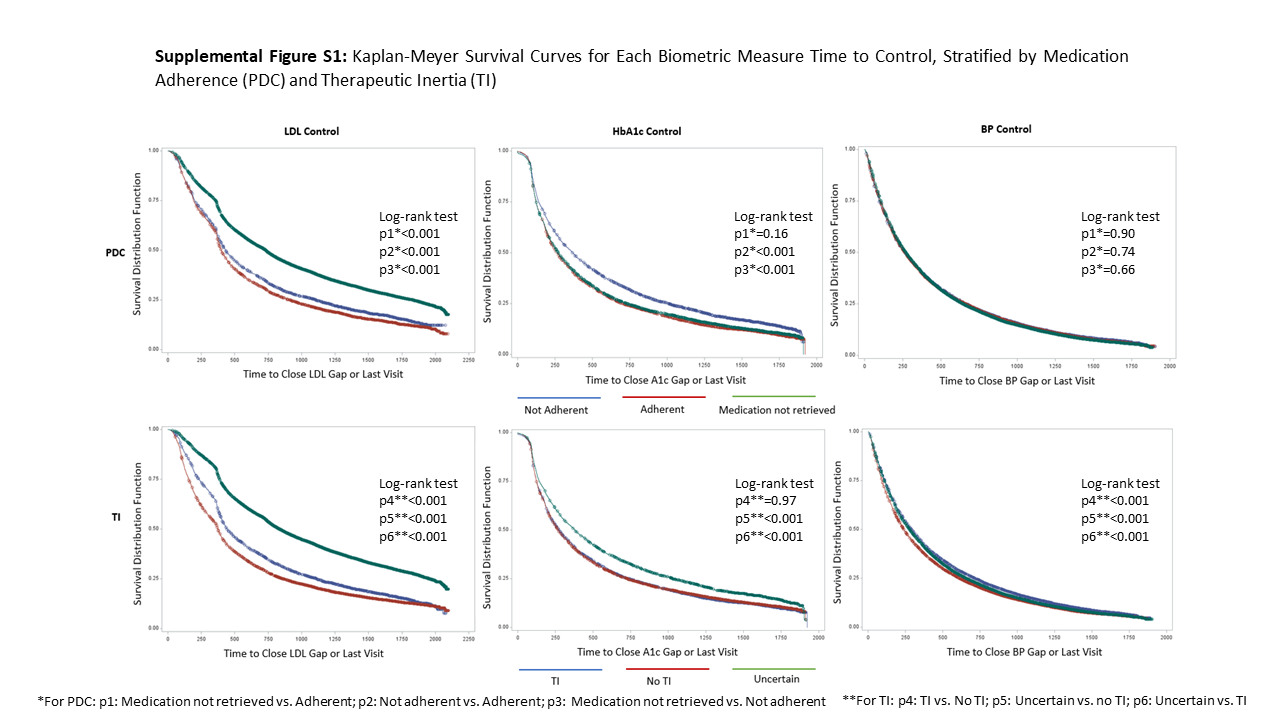

Supplement: Supplementary file 1 — Supplementary Information 1. [file 41598_2022_21916_MOESM1_ESM.png]
