# Supplementary material for: Medication non-adherence and therapeutic inertia independently contribute to poor disease control for cardiometabolic diseases
Source: Sci Rep. 2022 Nov 7;12:18936. doi: 10.1038/s41598-022-21916-8 (PMC9640683; doi:10.1038/s41598-022-21916-8)
Supplement: Supplementary file 2 — Supplementary Information 2. [file 41598_2022_21916_MOESM2_ESM.doc]

Supplementary Information

**Supplementary Table S1: Patient demographics and clinical information, outcomes for patients who met the inclusion criteria, stratified by cardiometabolic condition**

| **Patient demographics and Other factors** | **Hypertension Patients with BP gap at baseline**  **(N=59,087)** | **Diabetes Patients with HbA1c gap at baseline**  **(N=16,603)** | **Dyslipidemia patients with LDL gap at baseline (N=39,192)** |
| --- | --- | --- | --- |
| Age Group |  |  |  |
| 34-39 | 4.3% | 6.6% | 5.6% |
| 40-49 | 14.3% | 22.3% | 20.7% |
| 50-64 | 22.7% | 28.6% | 30.2% |
| 65-79 | 28.3% | 25.7% | 25.6% |
| 80+ | 30.4% | 16.9% | 17.8% |
| Charlson Score |  |  |  |
| 0 | 57.4% | 41.7% | 66.1% |
| 1-2 | 35.2% | 46.9% | 28.9% |
| 3+ | 7.4% | 11.4% | 5.0% |
| PDC status |  |  |  |
| Not adherent | 13.4% | 22.8% | 8.3% |
| Adherent | 38.7% | 37.8% | 13.8% |
| No medication retrieved | 47.9% | 39.4% | 77.9% |
| Race/Ethnicity |  |  |  |
| Hispanic | 9.9% | 17.4% | 10.4% |
| Non-Hispanic Asian | 10.1% | 15.4% | 15.0% |
| Black | 4.6% | 5.6% | 3.8% |
| Non-Hispanic Other | 11.0% | 13.4% | 12.4% |
| Non-Hispanic White | 64.4% | 48.2% | 58.4% |
| Sex |  |  |  |
| Female | 56.8% | 45.9% | 60.1% |
| BMI |  |  |  |
| <25 | 23.4% | 11.9% | 23.2% |
| 25-29 | 35.1% | 28.7% | 37.7% |
| 30-34 | 23.3% | 28.9% | 23.3% |
| 35+ | 17.9% | 30.1% | 15.5% |
| Missing | 0.4% | 0.5% | 0.3% |
| Smoking status |  |  |  |
| Never | 55.9% | 56.5% | 60.9% |
| Passive/quit | 38.1% | 36.7% | 33.0% |
| Unknown |  |  |  |
| Yes | 6.0% | 6.8% | 6.1% |
| Clinical Inertia |  |  |  |
| No | 32.9% | 39.1% | 26.8% |
| Yes | 18.6% | 42.8% | 9.6% |
| uncertain | 48.5% | 18.0% | 63.6% |
| Number of Cardiometabolic conditions |  |  |  |
| 1 | 25.0% | 1.7% | 20.2% |
| 2 | 49.4% | 18.0% | 48.4% |
| 3 | 25.7% | 80.2% | 31.4% |
| Diabetes before Dyslipidemia | N=31823 | N=16603 | N=14145 |
| Yes | 20.4% | 46.2% | 39.0% |
| Diabetes before Hypertension | N=17076 | N=16603 | N=22047 |
| Yes | 31.2% | 39.1% | 18.1% |
| Hypertension before Dyslipidemia | N=59087 | N=15217 | N=29452 |
| Yes | 29.0% | 32.9% | 39.1% |
| Time to close LDL gap  (n, medium, IQR) | N=9900*  414, 210-770 | N=3603***  395, 189-707 | 408, 214-758 |
| Time to close BP gap (n, medium, IQR) | N=53112  223, 92-494 | N=5327**  194, 84-452 | N=12380*  232, 93-518 |
| Time to close HbA1c gap (n, medium, IQR) | N=4978**  224, 115-517 | N=14252  224, 117-500 | N=3869***  235 (116-519) |
| Length with Dyslipidemia | (n=13817)  4.3 (1.3) | N=4635***  4.5 (1.2) | 4.2 (1.4) |
| Length with HTN | 4.8 (1.2) | N=5767**  5.2 (0.7) | N=5767**  5.2 (0.7) |
| Length with DM | 4.8 (1.3) | 4.7 (1.4) | 4.7 (1.4) |
| Baseline LDL level | N=13817*  140.7 (28.2) | N=4653***  127 (25) | 147.5 (30.3) |
| Baseline Systolic BP/Diastolic BP | N=59087  151 (12)/82 (11) | N=5767**  152.6 (12.7)/81.0 (10.8)) | N=13817*  151.9 (12.8)/82.3(10.5) |
| Baseline HbA1c | N=5767*  9.2 (1.4) | 9.3 (1.4) | N=4635  9.4 (1.4)*** |
| *Among those who had BP and LDL health gaps (n=13,817)  **Among those who had BP and HbA1c health gaps (n=5767)  ***Among those who had HbA1c and LDL-C health gaps (n=4635) | | | |

**Supplemental Table S2.** Hazard Ratio estimate from Cox regression models for each biometric measure comparing patients with multiple health gaps vs. a single health gap*

| **Variable** | **HR (95%CI)** | | |
| --- | --- | --- | --- |
|  | **BP control^†^** | **LDL-C control^‡^** | **HbA1c Control^§^** |
| HbA1c and BP gap vs. single gap | 0.99 (0.96-1.04) | - | 0.98 (0.94-1.03) |
| HbA1c and LDL gap vs. single gap | - | 0.98 (0.93-1.03) | 0.94 (0.89-0.98) |
| LDL and BP gap vs. single gap | 0.91 (0.89-0.93) | 0.96 (0.93-0.99) | - |
| Three gaps vs. single gap | 0.92 (0.86-0.95) | 1.15 (1.08-1.21) | 0.95 (0.90-1.0) |
| *Controlling for baseline demographic characteristics, corresponding medication adherence, therapeutic inertia, body mass index (BMI), Charlson comorbidity index (CCI), corresponding baseline biometric value  †, Among patients with BP gap, single gap means BP gap only  ‡, Among patients with LDL-C gap, single gap means LDL-C gap only  § Among patients with HbA1c gap, single gap means HbA1c gap only | | | |
